# Validation of dbEST-SSRs and transferability of some other solanaceous species SSR in ashwagandha [*Withania Somnifera* (L.) Dunal]

**DOI:** 10.1007/s13205-015-0295-4

**Published:** 2015-03-17

**Authors:** Eva K. Parmar, Ranbir S. Fougat, Chandni B. Patel, Harshvardhan N. Zala, Mahesh A. Patel, Swati K. Patel, Sushil Kumar

**Affiliations:** 1 Department of Agricultural Biotechnology, Anand Agricultural University, Anand, Gujarat 388 110 India; 2 Medicinal and Aromatic Plants Unit, Anand Agricultural University, Anand, Gujarat 388 110 India

**Keywords:** Ashwagandha, Cross-genera transferability, Expressed sequence tags, Genomic SSR, Medicinal plant, *Withania somnifera*

## Abstract

Cross-species transferability and expressed sequence tags (ESTs) in public databases are cost-effective means for developing simple sequence repeats (SSRs) for less-studied species like medicinal plants. In this study, 11 EST–SSR markers developed from 742 available ESTs of *Withania Somnifera* EST sequences and 95 SSR primer pairs derived from other solanaceous crops (tomato, eggplant, chili, and tobacco) were utilized for their amplification and validation. Out of 11, 10 EST–SSRs showed good amplification quality and produced 13 loci with a product size ranging between 167 and 291 bp. Similarly, of the 95 cross-genera SSR loci assayed, 20 (21 %) markers showed the transferability of 5, 27, 32, and 14.2 % from eggplant, chili, tomato, and tobacco, respectively, to ashwagandha. In toto, these 30 SSR markers reported here will be valuable resources and may be applicable for the analysis of intra- and inter-specific genetic diversity in ashwagandha for which till date no information about SSR is available.

## Introduction

Out of a large number of medicinal plants known in Indian medicine system, *Withania somnifera* (L.) Dunal or Indian Ginseng or ashwagandha is a valued medicinal plant with pharmaceutical and nutraceutical uses. It is a dicotyledonous plant of the family Solanaceae and widely used in traditional medical systems of India and Africa as adaptogens (physical and mental health promoter) or vitalizers (Dasgupta et al. 2014).

Despite its commercial value, there is a lack of improved varieties of *W. somnifera*, and crop improvement studies need to be taken up vigorously (Mir et al. 2013). Hence, to enhance the production and productivity of *W. somnifera* through breeding, information regarding genetic diversity is essential. There are reports where researchers have used phytochemicals like withanolides to analyze genetic diversity (Scartezzini et al. 2007; Tripathi et al. 2012). But the plant-to-plant variation in quantity and quality of active constituents is observed because environmental and edaphic conditions (Genotype × environment interaction) affect the active constitutions and activity profiles in medicinal plants (Oleszek et al. 2002). Similarly, studies based on morphometric data have been undertaken to analyze the nature and extent of genetic diversity in this important medicinal plant (Misra et al. 1998; Khatak et al. 2013). However, similar to phytochemicals, morphological studies are not highly reliable as these are also influenced by environmental effects. DNA markers are quite stable and not influenced by environmental conditions and therefore can be used to describe patterns of genetic variation and in authentication of plant—a critical issue in medicinal plants. In a previous study, many dominant marker systems like RAPD (Tripathi et al. 2012; Khatak et al. 2013), ISSR (Tripathi et al. 2012; Bamhania et al. 2013), SAMPL, and AFLP (Negi et al. 2006) have been employed in *W. somnifera*.

However, practically no sequence-based molecular markers and other genomic tools have been developed so far for *W. somnifera* molecular breeding. Recently, Gupta et al. (2013) detected simple sequence repeats (SSRs) in transcriptome data which is not still publicly available. Microsatellite markers (SSRs) are becoming the markers of choice due to their high robustness and polymorphism. However, the development of microsatellite markers is an expensive process. Recently, with the development of functional genomics, expressed sequence tags (ESTs) in a public sequence database provide a potential source of SSRs. Despite pharmacological importance, the transcriptomic and genomic data of *W. somnifera* are very limited and only 742 ESTs are available in the National Center for Biotechnology Information (NCBI) database. Even this limited database is not mined to develop useful SSRs. Therefore, due to limited ESTs in NCBI, it is highly valuable to investigate the transferability of SSR markers from other solanaceous species, where they are easily developed and evaluated, to *W. somnifera*. Consequently, SSRs with good transferability can be applied for comparative mapping and genomic synteny among genera. There still has been no report of the development of SSR markers from *W. somnifera*. Here, we report the designing and validation of EST–SSRs and transferability of some other solanaceous species SSR in *W. somnifera*.

## Materials and methods

### Plant materials and DNA isolation

A set of 12 genotypes of *W. somnifera* were used in the present study for the validation of SSR markers. Genomic DNA from genotypes was extracted from leaf samples collected from the experimental farm of Medicinal and Aromatic Plant Research Station, Anand Agricultural University, during May 2014. A total of 0.1 g leaf material was used for each genotype and DNA was isolated using DNeasy Plant Mini Kit (QIAGEN, Hilden, Germany) following the manufacturer’s instructions. The DNA concentration was estimated by agarose gel electrophoresis using DNA standard.

### Cross-species amplification of SSR primers

A total of 95 microsatellite primer pairs developed in other solanaceous crops (tomato, eggplant, chili, and tobacco) were procured to amplify SSR loci in *W. somnifera*. Polymerase chain reactions were performed in a final volume of 15 μl containing 1× PCR buffer, 1 μl MgCl_2_, 0.5 μl dNTPs, 1 μM of primers (10 pmol), 0.3 μl of Taq DNA polymerase (5 U/μl), and 20 ng of template DNA. Amplifications were carried out on a thermal cycler (Eppendorf, Germany) under the following conditions: initial denaturation at 94 °C for 3 min, followed by 35 cycles of 94 °C for 30 s, annealing temperature (Ta) recommended for the source-species amplification ±5 °C for 40 s, and 72 °C for 60 s with a final extension of 10 min at 72 °C. PCR products were visualized on 1.5 % (w/v) agarose gel using 1× TBE buffer and stained with ethidium bromide, then visualized and photographed using gel documentation system (Bio-Rad, Hercules, California). The allele sizes were calculated by comparing with 50-bp DNA ladder (GeNeiTM, Bangalore, India).

### Database search and EST-SSR primer designing

*W. somnifera* ESTs were acquired by searching NCBI (www.ncbi.nlm.nih.gov; up to August 2014). The downloaded sequences were obtained in FASTA format for sequence assembly and SSR analysis. dbEST has redundancy in EST sequences. In order to remove the redundancy, ESTs were assembled into unigenes using CAP3 assembler (Huang and Madan 1999). Non-redundant FASTA-formatted sequences were submitted to the high-throughput web application BatchPrimer3 (You et al. 2008; http://probes.pw.usda.gov/cgi-bin/batchprimer3/batchprimer3.cgi) to screen perfect microsatellite repeat motifs and to design corresponding PCR primers. The primers were designed with a length of 17–24 bp, an annealing temperature of 50–60 °C, and the product sizes ranging from 100 to 500 bp. PCR for EST-SSR was performed as described in the previous section.

### Data analysis

The amplified fragments were classified into four classes based on the signal intensity as described by Kuleung et al. (2004): (1) strong signal and easy to score, (2) weaker signal but able to score, (3) very weak signal and difficult to score, and (4) no signal. Classes 1 and 2 were considered positive for amplification, whereas classes 3 and 4 were considered negative for amplification.

## Results and discussion

### Cross-species transferability

**Fig. 1:**
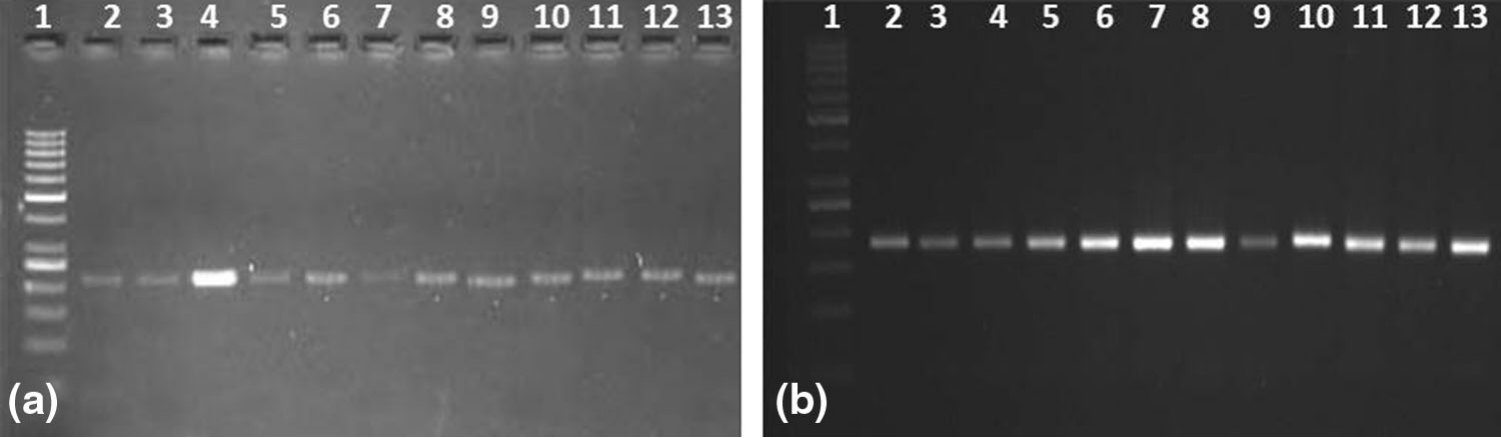
Amplification profile of (**a**) CaES3962 SSR through cross-species amplification and (**b**) WS_SSR08, a dbEST-SSR, in 12 genotypes of *Withania somnifera*; *lane 1* 100-bp ladder DNA marker; *lane 2*–*13* AWR 1, AWR 2, AWR 3, AWR 4, AWR 5, AWR 6, AWS 3-49, AWS 21, AWS 54, AWS 60/1, AWS 62, and AWS 19, respectively

SSR markers have been exploited extensively for genotypes identification, plant variety protection, and as anchor markers in linkage mapping and in marker-assisted breeding (Sukanya et al. 2011). But they are expensive to identify and develop and presently not available in *W. somnifera*, a medicinal crop of commercial importance. As development of SSR is time consuming, labor intensive, and costly, it is highly valuable to investigate the transferability of SSR markers among related species/genera. SSRs have been developed and widely utilized in other Solanaceous crops (tobacco, tomato, pepper, eggplant, etc.), and there have been several reports on the transferability of SSR markers in or across genera among different crops (Mishra et al. 2011; Rai et al. 2013; Kumar et al. 2014). In the present study, the transferability of SSR was analyzed through the screening of primers from other solanaceous species to *W. somnifera*. A total of 95 SSR primers from eggplant (20), chili (26), tomato (28), and tobacco (21) to *W. somnifera* were assessed for their ability to amplify across the genera. Out of which 95 primer pairs tested, 20 primers amplified and gave cross-genera transferability. The result indicated that 1 (5 %) from eggplant, 7 (27 %) from pepper, 9 (32 %) from tomato, and 3 (14.2 %) from tobacco are transferable to *W. somnifera* (Table 1; Fig. 1a). Our results are in accordance with many previous studies that have shown intergeneric transferability of SSR, for instance carrot to cumin (38 %; Kumar et al. 2014), guava to clove (73.9 %; Rai et al. 2013), *Setaria italica* to six grass species (74 %; Gupta et al. 2012). The variable degree of transferability of SSR markers can be attributed to sequence conservation, and the results of the present work support the perception that the members of Solanaceae are genetically similar and there is a possibility of cross-amplification at genetic loci between different species. However, the rate of cross-amplification was low (21 %) as compared to the previous report in *W. somnifera* (Sukanya et al. 2011). It may be due to that cross-species amplification of microsatellite markers from source to target species is generally negatively correlated with evolutionary divergence (Maduna et al. 2014). Apart from the source–target species’ evolutionary distance, other factors, such as mutations in microsatellite flanking sequences, source species of SSRs, and number and type of SSR screened, may affect the success rate of cross-species amplification. Our results provide an undoubted confirmation for the potential transferability of SSRs across genera. The amplicon size for each primer observed in this study is almost similar to that reported in source species (Table 1). However, the transferable rates generated by these primers are medium and provide an opportunity for studying *W. somnifera*. The 13 microsatellites produced bright and dense bands on amplification. However, only seven (35 %) SSRs were found to be polymorphic.

**Table 1:** Result of amplified cross-species-transferred primers with their sequences

Primer name	Primer sequence (5′–3′)	Tm (°C)	Product size^a^	Polymorphism	Quality^b^	Specificity^c^
A1773078F^#^	GATGGACACCCTTCAATTTATGGT	60.1	110 (145–190)	No	+	+
A1773078R^#^	TCCAAGTATCAGGCACACCAGC					
AW034362F^#^	CCGCCTCTTTCACTTGAAC	60.1	135 (130–175)	No	+	+
AW034362R^#^	CCAGCGATACGATTAGATACC					
SSR 9F^#^	CCCTTTGCAAGTTCTTCTTCA	55.9	175 (175–250)	No	++	+
SSR 9R^#^	TTCATGAGCCAACATAGGAGG					
SSR 13F^#^	GGGTCACATACACTCATACTAAGGA	60.1	125/130 (102)	Yes	++	+
SSR 13R^#^	CAAATCGCGACATGTGTAAGA					
SSR 20F^#^	GAGGACGACAACAACAACGA	60.2	120/140 (157)	Yes	+	+
SSR 20R^#^	GACATGCCACTTAGATCCACAA					
SSR 22F^#^	GATCGGCAGTAGGTGCTCTC	60.2	185/200 (218)	Yes	++	+
SSR 22R^#^	CAAGAAACACCCATATCCGC					
SSR 32F^#^	TGGAAAGAAGCAGTAGCATTG	55.9	205 (187)	No	++	+
SSR 32R^#^	CAACGAACATCCTCCGTTCT					
SSR 63F^#^	CCACAAACAATTCCATCTCA	53.2	285 (250)	No	++	+
SSR 63R^#^	GCTTCCGCCATACTGATACG					
SSR 76F^#^	ACGGGTCGTCTTTGAAACAA	55.3	210/200 (199)	Yes	+	+
SSR 76R^#^	CCACCGGATTCTTCTTCGTA					
CaES1169F^@^	CCTGTTGAACGTCTTGCCTT	55.3	725/235 (206)	Yes	++	−
CaES1169R^@^	TTCTTCTTGCTCCCTTTGGA					
CaES3962F^@^	GGAAGAAAAAGCCTGTTCCC	53.2	235 (218)	No	++	+
CaES3962R^@^	TCCCTGCATCAAACATTGAA					
CaES4072F^@^	CAGCACGCTTGCTAATTCAA	55.3	125/130 (121)	Yes	+	+
CaES4072R^@^	AGCAGGCTTGGAATCCACTA					
CaES4787F^@^	CCAAACGAGTCCCACCTAGA	57.3	130 (132)	No	++	+
CaES4787R^@^	TTAGGTCCCGGACAAGAAGA					
CaES4883F^@^	CTTTTCGTTTTTGGGTGGAA	53.2	130 (212)	No	++	+
CaES4883R^@^	GAGGGGCCGTCATAATTACA					
CaES5301F^@^	TGTAAAATCCGGGTGGAAGA	53.2	640/650 (624)	Yes	++	+
CaES5301R^@^	TTTTCCATGGTTTCAAAGGC					
CaMS142F^@^	GAGCGCTTAAGTGGTCATAGG	55.3	150 (241)	No	++	–
CaMS142R^@^	CTACAACGCCCCAAAACAAT					
PT20168F^$^	TAGGTTCCTCCCTTCTCG	50.0	140 (149)	No	+	+
PT20168R^$^	CCCGATCCAAAAAGAGAT					
PT30142F^$^	TCGCTTAACTGTTGCTTCCC	61.0	170/200/215 (177)	No	+	−
PT30142R^$^	CTGAGGAACTCCAAACGCTC					
PT30375F^$^	TCCTCTACCCAACGTCAAGAA	61.0	150 (230)	No	++	+
PT30375R^$^	GGCAAACCAGCTAGCACAT					
SM36F^^^	AGCACCAGGACAATGAATAC	52.1	300/320 (231)	No	++	+
SM36R^^^	CCATTTCTTTCTCGACCTTA					

^#^Source species: tomato

^@^Source species: chili

^$^Source species: tobacco

^^^Source species: egg plant

^a^Value in parenthesis is amplicon size in source species

^b^++ = Strong signal and easy to score; + = weaker signal but able to score

^c^+ = Amplified product of a similar size (within 100 bp) to that of source species; − = amplified product not of similar size

### Designing and validation of EST-SSR

Plant genome and transcriptome sequencing efforts have been centered on mostly economically important crops especially in the last decade. However, in present genomics era, due to the development of high-throughput next-generation sequencing platforms, a race for sequencing the genomes and transcriptomes of life-saving medicinal plants is underway. Out of ~370,000 known plant species, only ~80,000 species are represented by at least a single GenBank entry (Mishra 2014), of which medicinal plant genomics/transcriptomics have been less focused. ESTs, an output of transcriptomics, are often represented by redundant cDNA sequences making it difficult to analyze them effectively for SSRs (Joshi et al. 2011). To overcome this problem in the present study, the CAP3 program was used. The reduction in redundancy is used as a measure of degree of overlapping between EST sequences. The objective was the elimination of redundancy in EST sequences and arriving at a contiguous sequence (contigs) which can be used for the analysis of SSRs. CAP3 is a commonly used program, which identifies overlapping sequences and generates contigs with consensus sequences.

Till date, there are only 742 ESTs available for *W. somnifera* in NCBI database. These ESTs were retrieved and scanned for Class I microsatellite repeats and to design EST–SSR markers. Mono-SSRs were not considered since they do not serve as important molecular markers. The search criteria were kept low to maximize the SSR discovery. After trimming of poly A and poly T tails, rest of the sequences were clustered and assembled into a non-redundant dataset of 79 unique gene sequences (54 contigs and 25 singlets). Scanning of Class I microsatellites in this non-redundant dataset through BatchPrimer3 could reveal only 11 unique SSR-containing sequences. The frequency of EST–SSRs (1.4 %) identified in the present study is much lower compared to that identified in the earlier study in medicinal plant periwinkle (*Catharanthus roseus*) (Mishra et al. 2011) but in accordance with rose (2.4 %; Gupta et al. 2010). These results indicate that the frequency of SSRs in EST sequences may vary among different plant species, and this difference may be resulted from the number and length of ESTs.

The validation of the designed EST-SSR primers was carried out by the PCR amplification method. The primers developed were amplified on the 12 *W. somnifera* genotypes (Fig. 1b). The amplification of 10 primers out of 11 primers was noticed. Among all the amplified 90 % of products were found to be bright and scorable. After optimization, the seven primer pairs (70 %) amplified the expected products. Of the ten primer pairs, 6 (60 %) were polymorphic, while the others (40 %) were monomorphic. The primer sequences and amplicon result for these 10 loci are presented in Table 2. SSR markers derived from cross transferability are less polymorphic than ESTs-SSR, due to the fact that there is a significant conservation of marker among genera. A total of 13 alleles were detected in 10 loci, with an average of 1.3 per locus.

**Table 2:** Result of designed dbEST-SSR markers with their sequences

Primer name	Sequence (5′–3′)	GC (%)	Tm (°C)	Motif	Motif length	Product size (bp)	Polymorphism
WS_SSR01F	TCTGAATGACTCCCATCTTTTG	40.91	57.0	CATCAC	6	510/520	Yes
WS_SSR01R	TGGTGATGGTGATGTTGATGT	42.86					
WS_SSR02F	TGGGATGAACACAGACATCAA	42.86	57.0	CATCAC	6	250/260	Yes
WS_SSR02R	CTTGGAGGTGAACTAGCAAGAA	45.45					
WS_SSR03F	AGTCCCTCAAGGCCAAGAAA	50.00	57.0	GCA	3	220	No
WS_SSR03R	GCGGATCAGGAAATAGACGAA	47.62					
WS_SSR04F	TATTTCCTGATCCGCTCAATC	42.86	56.2	TGGGG	5	290/300	Yes
WS_SSR04R	TTTCAGAGAACCAACAAGTCCA	40.91					
WS_SSR06F	TGTTAAGGGTGATGCAGGAGA	47.62	57.0	CAG	3	150/170/190	Yes
WS_SSR06R	CATACACAAACCAAGCCCTAAA	40.91					
WS_SSR07F	GGATGCTATTGAAGCGATGAA	42.86	64.0	GGT	3	780	No
WS_SSR07R	ATATCCACCACCACCACCAC	55.00					
WS_SSR08F	GTGATGTTGGATACGGTGGAG	52.38	57.0	GGT	3	185/190	Yes
WS_SSR08R	CGGAACTTGAACAAAACTTGGA	40.91					
WS_SSR09F	GCAATTTCCTCTGAGTTTGGT	42.86	57.0	AAG	3	770/800	Yes
WS_SSR09R	CCTCTGTTTTCTCTTCTTCCTTG	43.48					
WS_SSR10F	CAGAGGACACATCAGTTCCAGTT	47.83	57.0	ACC	3	180	No
WS_SSR10R	TCCTTTCTTCTCTCTCCCCTCT	50.00					
WS_SSR11F	GAGGACACGTCAGTTCCAGTT	52.38	57.0	ACC	3	185	No
WS_SSR11R	TCCTTTCTTCTCTTTCCCCTCT	45.45					

## Conclusion

The polymorphism and transferability analysis of SSR markers indicated the value of developed markers. The applicability of cross-genera amplification of solanaceous SSR provides a good opportunity for studying *W. somnifera*. The new set of six polymorphic EST–SSR loci will enable the characterization of population genetic diversity and structure throughout the species in conjunction with cross-transferable SSRs in *W. somnifera* for which till date no information about EST-derived as well as genomic SSR is available.
